# Prediction of new thermodynamically stable aluminum oxides

**DOI:** 10.1038/srep09518

**Published:** 2015-04-01

**Authors:** Yue Liu, Artem R. Oganov, Shengnan Wang, Qiang Zhu, Xiao Dong, Georg Kresse

**Affiliations:** 1 Department of Geosciences, State University of New York, Stony Brook, NY 11794, USA; 2 Moscow Institution of Physics and Technology, 9 Institutskiy Lane, Dolgoprudny City, Moscow Region 141700, Russia; 3 School of Materials Science, Northwestern Polytechnical University, Xi'an 710072, China; 4 School of Physics and MOE Key Laboratory of Weak-Light Nonlinear Photonics, Nankai University, Tianjin 300071, China; 5 University of Vienna, Faculty of Physics and Center for Computational Materials Science, Sensengasse 8/12, A-1090 Wien, Austria

## Abstract

Recently, it has been shown that under pressure, unexpected and counterintuitive chemical compounds become stable. Laser shock experiments (A. Rode, unpublished) on alumina (Al_2_O_3_) have shown non-equilibrium decomposition of alumina with the formation of free Al and a mysterious transparent phase. Inspired by these observations, we have explored the possibility of the formation of new chemical compounds in the system Al-O. Using the variable-composition structure prediction algorithm USPEX, in addition to the well-known Al_2_O_3_, we have found two extraordinary compounds Al_4_O_7_ and AlO_2_ to be thermodynamically stable in the pressure ranges 330-443 GPa and above 332 GPa, respectively. Both of these compounds at the same time contain oxide O^2−^ and peroxide O_2_^2−^ ions, and both are insulating. Peroxo-groups are responsible for gap states, which significantly reduce the electronic band gap of both Al_4_O_7_ and AlO_2_.

Aluminum and oxygen are among the most abundant elements in the universe. Their only stable compound, alumina Al_2_O_3_, is widely used due to its mechanical properties (e.g. as an abrasive material) and due to its very wide band gap (for example, as an optical window material in shock-wave experiments).

Al_2_O_3_ in the corundum structure (space group 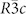) is an important mineral in the Earth's crust. Alumina is easily incorporated into many silicates and significantly affects their physical properties^1^,^2^. Several phase transitions have been theoretically predicted and experimentally confirmed to occur under pressure. It was shown that corundum transforms to the Rh_2_O_3_(II)-type structure (space group *Pbcn*) at ~80 GPa^3^,^4^ and to the CaIrO_3_-type phase (“post-perovskite”, space group *Cmcm*) above 130 GPa^5^,^6^. In 2007, Umemoto et al.^7^ predicted a further phase transition to a U_2_S_3_-type polymorph (space group *Pnma*) at ~370 GPa.

Given the high degree of ionicity of the Al-O bond (due to the large electronegativity difference, 1.8 on the Pauling scale), and the only possible oxidation states being +3 and −2 for Al and O, respectively, the only possible stable compound seems to be Al_2_O_3_. Of course, one can also imagine a peroxide with composition Al_2_(O_2_)_3_ = AlO_3_, but such a compound has never been reported.

While no other stable oxides are known, there is evidence for metastable AlO_2_, which was shown to form by an interfacial reaction in the presence of a kinetic constraint during diffusion-bonding of Pt and α-Al_2_O_3_. Raman spectroscopy has provided strong evidence for the presence of AlO_2_^8^, which formed after heating for 24 hours in the temperature range 1200–1400°C. AlO_2_ is a “peroxide oxide”, i.e. contains peroxide (O_2_^2−^), and oxide (O^2−^) ions. It was not clear whether AlO_2_ or other unusual oxides are stable at any pressure-temperature conditions.

Very recently, it has been shown that even in seemingly extremely simple systems, such as Na-Cl, totally unexpected compounds (Na_3_Cl, Na_2_Cl, Na_3_Cl_2_, NaCl_3_ and NaCl_7_) become stable under pressure – these have been predicted using evolutionary crystal structure prediction method USPEX and verified by experiments^9^. If such unusual compounds exist in the “trivial” Na-Cl system, one can expect similarly unusual compounds in nearly any other system under pressure. Here we test this hypothesis on the Al-O system, and indeed predict that Al_4_O_7_ and AlO_2_ become thermodynamically stable under high pressure.

## Computational Methodology

To predict stable Al-O oxides and their structures, we used the evolutionary algorithm USPEX^10^,^11^,^12^ in its variable-composition mode^13^ at pressures 0, 50, 100, 150, 200, 300, 400, 500 GPa. The reliability of USPEX has been demonstrated many times before – e.g. Ref. 9, 14–18. Modern methods have shown remarkable power to predict novel unexpected compounds – e.g. in the Na-Cl^9^, Mn-B^19^, Mg-C^20^ and Na-Si^21^ systems. Stable compositions were determined using the convex hull construction: a compound is thermodynamically stable when its enthalpy of formation from the elements and from any other compounds is negative. Enthalpy calculations and structure relaxations were done using density functional theory (DFT) within the Perdew-Burke-Ernzerhof (PBE) generalized gradient approximation (GGA)^22^, as implemented in the VASP code^23^. These calculations were based on the all-electron projector-augmented wave (PAW) method^24^ and plane wave basis sets with the kinetic energy cutoff of 600 eV and uniform Γ-centered k-point meshes with reciprocal-space resolution of 2π*0.02 Å^−1^. The first generation of structures/compositions was produced randomly with the use of space group symmetries (using algorithm^12^); the lowest-fitness 60% of the structures/compositions were allowed to produce child structures/compositions (fitness being defined as the difference between enthalpy of the structure and the convex hull). Initial structures were allowed to have up to 20 atoms in the unit cell, but this range was allowed to change in subsequent generations as a result of evolution. Child structures/compositions were created in the following manner: 20% by random symmetric generator, 40% by heredity, 20% by softmutation, and 20% by atomic transmutation. In this work, we first performed searches in the entire Al-O system with up to 20 atoms/cell, and have found only Al_2_O_3_ and oxygen-enriched phases Al_4_O_7_ and AlO_2_. Then we did additional focused searches in a narrower compositional range Al_2_O_3_-O, and obtained the same result.

After USPEX predictions, we selected structures on the convex hull and close to it, and relaxed them carefully at pressures 0, 10, …, 520 GPa. These calculations have confirmed stability of three oxides – well-known Al_2_O_3_ and non-classical AlO_2_ and Al_4_O_7_. For these compounds, we also computed their electronic band structures. For accurate estimates of the band gaps, we have used the HSE hybrid functional^25^. Phonon frequencies throughout the Brillouin zone were calculated using the finite displacement approach as implemented in the Phonopy code^26^,^27^, and these calculations confirmed that these phases are dynamically stable at pressure ranges where our enthalpy calculations predict their thermodynamic stability.

## Results

### Stable compounds in the Al-O system

At all pressures in the range 0–500 GPa, the known compound - Al_2_O_3_ - is found to be thermodynamically stable. In agreement with previous works we find the same sequence of phase transitions – from corundum to the Rh_2_O_3_(II)-type structure at 100 GPa, then to the CaIrO_3_-type structure at 130 GPa, and then to the U_2_S_3_-type phase at 394 GPa.

**Figure 1:**
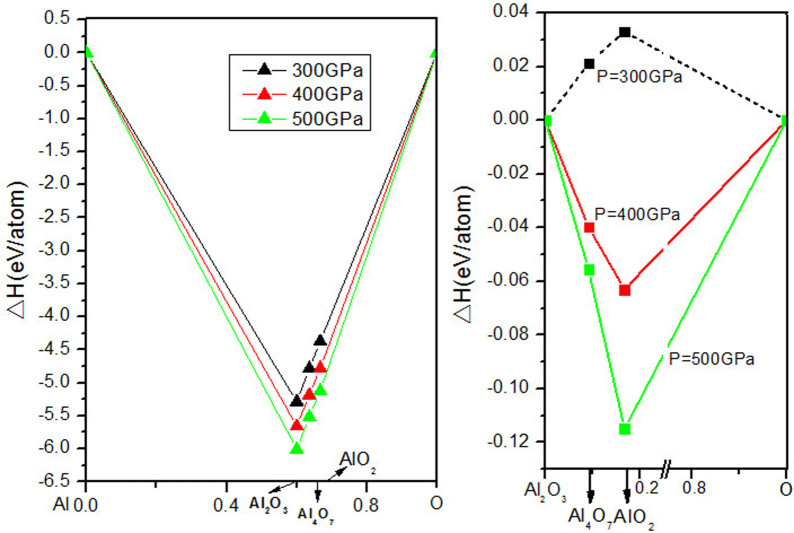
Thermodynamics of the Al-O and Al_2_O_3_-O systems. For the end members we used the theoretically predicted lowest-enthalpy structures from this work and Ref. 27.

**Figure 2:**
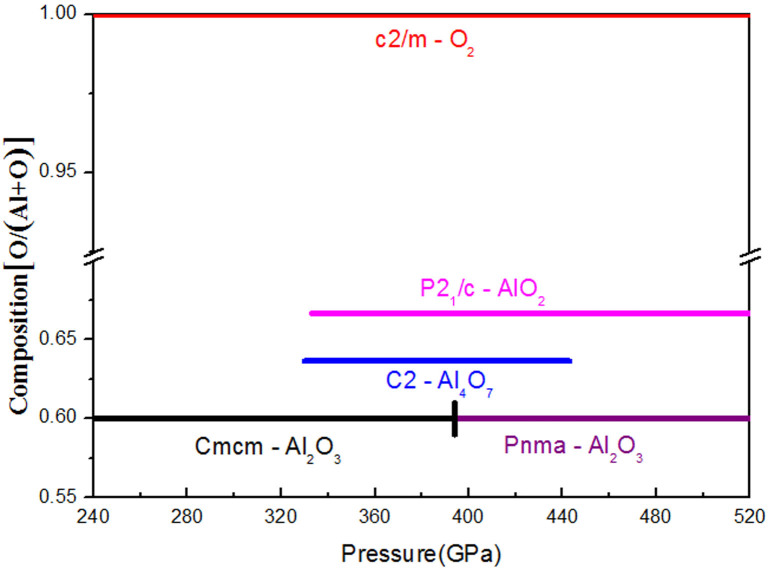
Pressure-composition phase diagram of the Al_2_O_3_-O system.

The computed thermodynamics of Al-O compounds are shown in Fig. 1. Al_4_O_7_ and AlO_2_ begin to show competitive enthalpies of formation at pressures above 300 GPa and have stability fields at 330–443 GPa and at >332 GPa, respectively. From Fig. 1, one can see that at 500 GPa the enthalpy of formation of AlO_2_ from Al_2_O_3_ and O is impressively negative, −0.12 eV/atom. The predicted pressure-composition phase diagram is shown in Fig. 2. To assess the effect of temperature, we performed quasi-harmonic free energy calculations for AlO_2_, Al_2_O_3_ and O. We found that temperature and zero-point vibrations stabilize AlO_2_ and expand its stability field: at T = 300 K it becomes stable at 321 GPa, at T = 2060 K at 300 GPa, at T = 3200 K at 280 GPa.

### Structures of stable compounds: Al_4_O_7_ and AlO_2_

Structures of the stable phases of Al_2_O_3_ have been discussed elsewhere, so here we focus only on the new compounds, Al_4_O_7_ and AlO_2_. Each of these compounds has only one stable structure up to 500 GPa, and both contain at the same time oxide O^2−^ and peroxide [O-O]^2−^ anions, i.e. both can be described as “oxide peroxides”. At normal conditions, the O-O bond lengths are^28^ 1.21 Å in the O_2_ molecule, 1.28 Å in the superoxide O^2−^ ion, and 1.47 Å in the peroxide O_2_^2−^ ion. In Al_4_O_7_ the O-O bond length is 1.43 Å at 400 GPa, in AlO_2_ it is 1.38 Å at 500 GPa – clearly indicating the presence of peroxide-ions.

**Figure 3:**
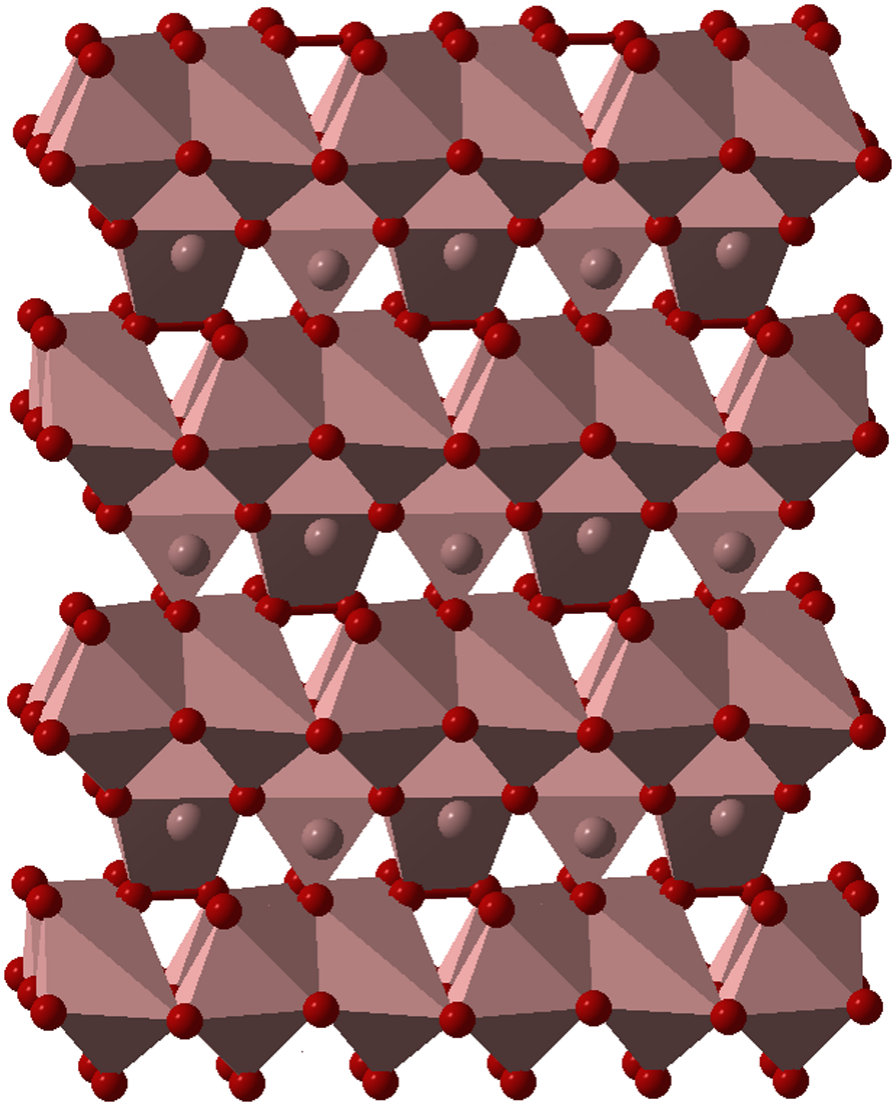
Structure of Al_4_O_7_ at 400 GPa. The structure contains 7- and 8-coordinate Al atoms (coordination polyhedra are shown). Some layers of the structure contain only oxide O^2−^ ions, other layers contain both oxide O^2−^ and peroxide O_2_^2−^ ions. Peroxo-ions have two O atoms connected by a bond; the O-O bond length is 1.43 Å.

**Figure 4:**
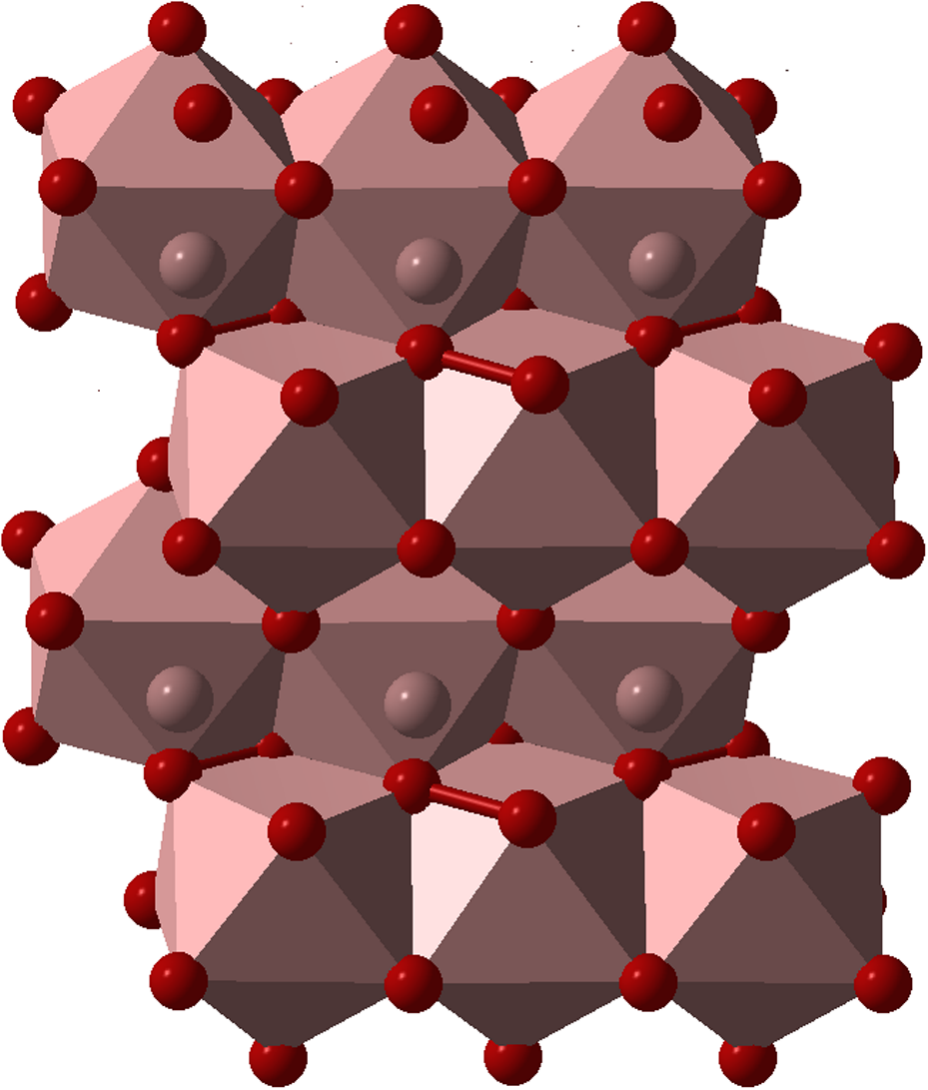
Structure of AlO_2_ at 500 GPa. Al atoms are in the 9-fold coordination (coordination polyhedra are shown). Oxide O^2−^ and peroxide O_2_^2−^ ions are arranged in alternating planes. Peroxo-ions have two O atoms connected by a bond; the O-O bond length is 1.38 Å.

The chemical formulas of these compounds can be obtained from Al_2_O_3_ by consecutive replacement of O^2−^ by O_2_^2−^ (which has the same charge): taking two formula units Al_4_O_6_ and replacing O_2_→O_2_^2−^, we obtain Al_4_O_5_(O_2_) = Al_4_O_7_, and doing the same replacement again, we obtain Al_4_O_4_(O_2_)_2_ = AlO_2_. These are indeed the structural formulas: Al_4_O_5_(O_2_) for Al_4_O_7_, and Al_4_O_4_(O_2_)_2_ for AlO_2_. These structures are shown in Figs. 3 and 4, and their parameters are given in Table 1.

## Discussion

### Properties of the new phases

**Figure 5:**
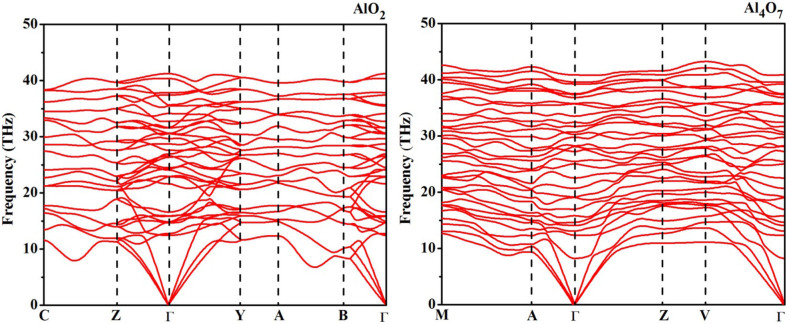
Phonon dispersion curves of AlO_2_ (left) and Al_4_O_7_ (right) at 400 GPa.

Phonon dispersion curves of Al_4_O_7_ and AlO_2_, computed at 400 and 500 GPa, respectively, are shown in Fig. 5. Both phases are dynamically stable and display a continuum of phonon energies, i.e. absence of decoupled O-O vibrational modes of peroxo-groups, because at high pressure Al-O modes have frequencies comparable to O-O modes. At the same time, in the electronic structure, there are clearly defined dispersive bands of peroxo-groups, and these play an important role, as we discuss below. Both phases are dynamically and mechanically stable, as shown by their computed phonons, elastic constants, and evolutionary metadynamics^29^ simulations, also enabled in the USPEX code and allowing one to explore possible phase transitions. We have confirmed that there are indeed no distortions or modulations that could lead to more stable structures.

**Figure 6:**
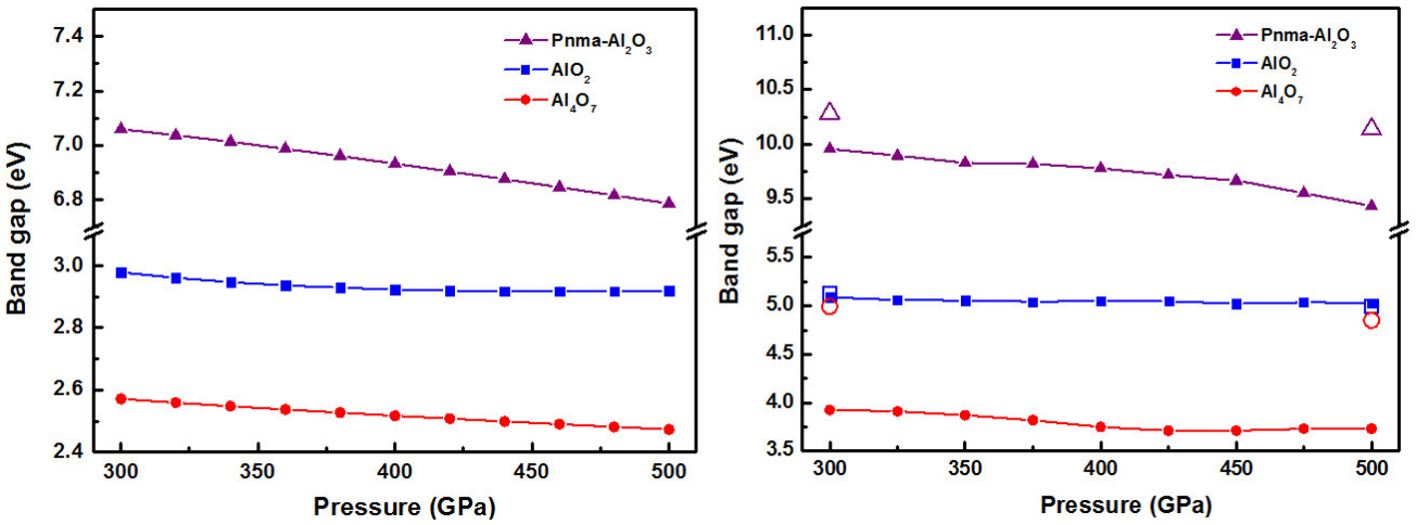
Band gaps of AlO_2_, Al_4_O_7_ and Al_2_O_3_ as a function of pressure with (a) PBE functional, (b) HSE functional and GW method (filled symbols - HSE results, open symbols - GW results).

All the predicted phases are insulating and show very distinct electronic structure compared with Al_2_O_3_. At 400 GPa, the computed DFT band gaps are 6.93 eV for *Pnma*-Al_2_O_3_, 2.51 eV for Al_4_O_7_, 2.92 eV for AlO_2_. We recall that DFT calculations significantly underestimate band gaps, while hybrid functionals and GW approximation give much better band gaps, typically within 5-10% of the true values. Fig. 6 shows band gaps as a function of pressure, computed using the GGA (PBE functional), hybrid HSE functional^25^ and GW approximation^30^,^31^; one can see that GGA band gaps are ~30% underestimates; HSE band gaps practically coincide with the most accurate GW values for AlO_2_, but are 0.2–1.1 eV lower for Al_2_O_3_ and Al_4_O_7_. At all levels of theory, Al_4_O_7_ and AlO_2_ come out to have band gaps ~2 times lower than the band gap of Al_2_O_3_.

**Figure 7:**
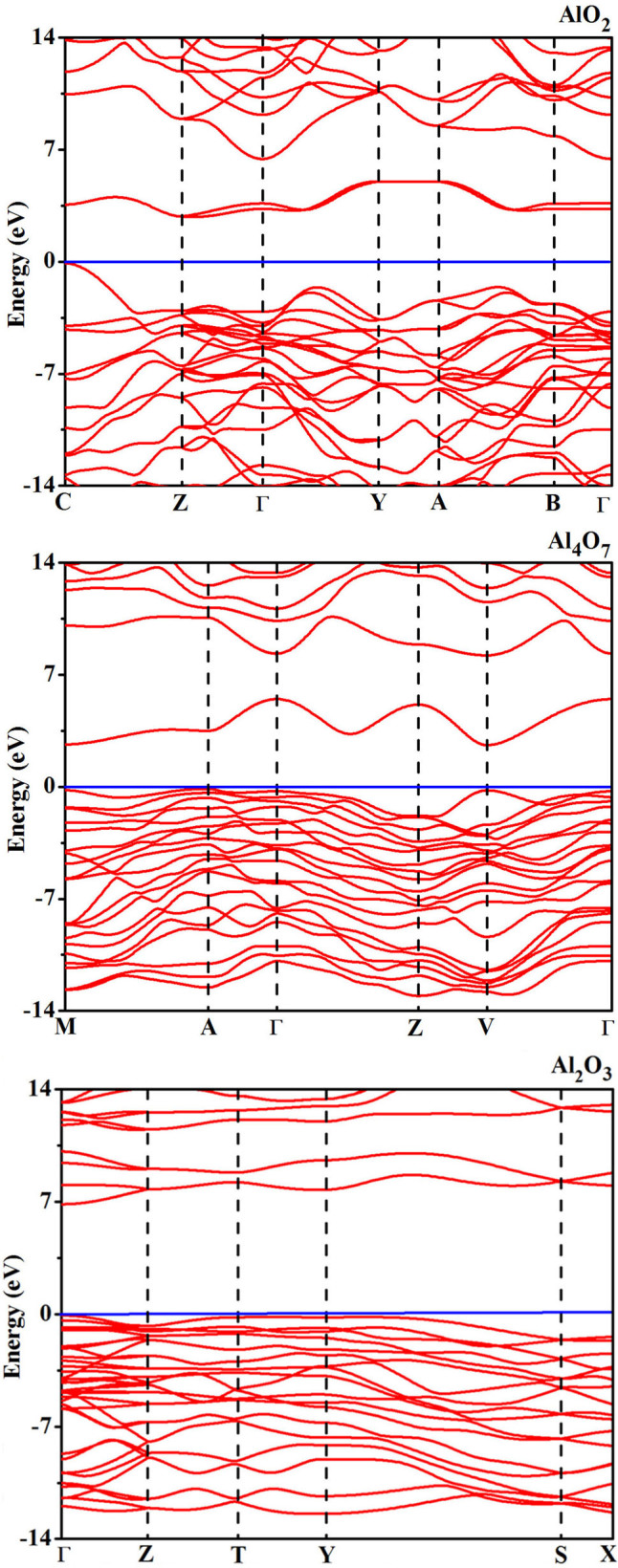
Band structures of AlO_2_, Al_4_O_7_ and Al_2_O_3_ at 400 GPa.

**Figure 8:**
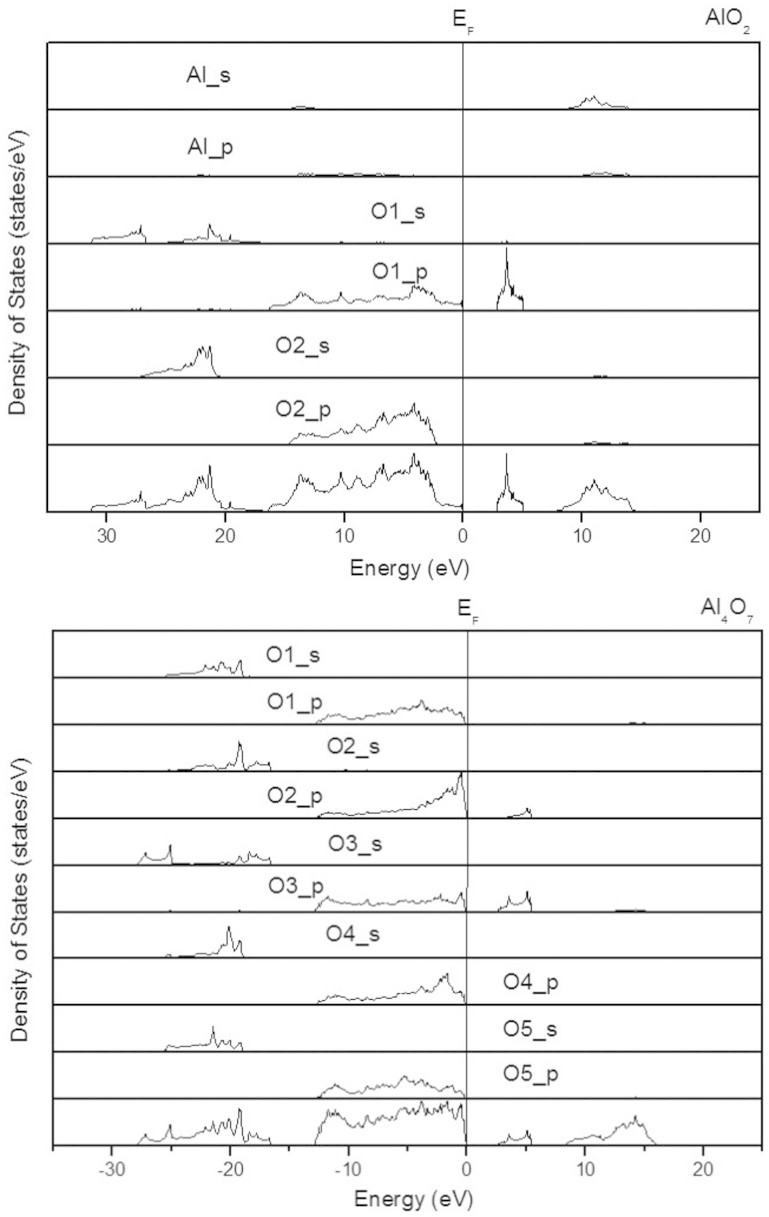
Projected electronic densities of states of AlO_2_ and Al_4_O_7_. (a) AlO_2_: O1 is a peroxide site and O2 is an oxide ion; (b) Al_4_O_7_: O2 and O3 are atoms from two different peroxide ions, and O1, O4, O5 are oxide sites.

This band gap reduction for Al_4_O_7_ and AlO_2_ originates from the additional low conduction band in the middle of the band gap. Our calculations (Fig. 7) show that these low conduction bands can be unequivocally assigned to the peroxo-groups. In both Al_4_O_7_ and AlO_2_, both gap states - the HOMO (highest occupied molecular orbital) and LUMO (lowest unoccupied molecular orbital) - come from peroxo-groups. Together with low compressibility of the peroxo-groups (between 300 GPa and 500 GPa, the O-O distance changes from 1.37 to 1.38 Å and from 1.46 to 1.42 Å in AlO_2_ and Al_4_O_7_, respectively), this explains why the band gaps of Al_4_O_7_ and especially AlO_2_ are practically independent of pressure in a wide pressure range (Fig. 6). As Fig. 8 shows, projected densities of states show only small contributions from Al, thus indicating a high degree of ionicity. Indeed, Bader charges^32^ are +2.44 of Al, −0.83 of O1 (peroxide anion) and −1.61 of O2 (oxide anion) in AlO_2_ at 400 GPa.

While the band gaps computed by DFT (PBE functional) are, as expected, significantly underestimated, the energetics are accurate. We have tested this by computing the energy and enthalpy of the reaction
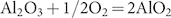
using the combined exact exchange (EXX) and random phase approximation (RPA) technique^33^,^34^,^35^. At 300 GPa we obtained the following energies (enthalpies) for this reaction: 0.1727 eV/atom (−0.0113 eV/atom) for the RPA+EXX method and 0.1782 eV/atom (0.0330 eV/atom) for PBE. At 500 GPa the results are 0.1284 eV (−0.1200 eV/atom) for RPA+EXX and 0.1371 eV (−0.1150 eV/atom) for PBE. These calculations fully confirm our findings and give additional insight:

1. In both PBE and EXX+RPA the new compounds are stabilized by the P*V-term in the free energy, rather than by the internal energy. This originates from the low packing efficiency in elemental oxygen, which remains a molecular solid in the entire pressure range studied here. For this reason we can expect increased reactivity of oxygen, and stabilization of oxygen-rich compounds (such as peroxides) at high pressures.
2. Results of the PBE and EXX+RPA are quantitatively similar, especially at 500 GPa, where the difference is only 5 meV/atom.
3. At the EXX+RPA level of theory the new compounds predicted here are even more stable than at the PBE level.

## Conclusions

Systematic search for stable compounds in the Al-O system at pressures up to 500 GPa revealed two new stable compounds (AlO_2_ and Al_4_O_7_); their stability fields are above 332 GPa and in the range 330–443 GPa, respectively. Our analysis reveals that insulating compounds AlO_2_ and Al_4_O_7_ exhibit significantly ionic character, both contain peroxide [O-O]^2−^ and oxide O^2−^ anions and therefore belong to the exotic class of “peroxide oxides”. Electronic levels of the peroxo-groups form gap states (“low conduction band”) that lead to a twofold lowering of the band gap relative to Al_2_O_3_. Our preliminary results show that the formation of peroxo-ions and stabilization of peroxides under pressure occur in many oxide systems, and this phenomenon may play an important role in planetary interiors, with their high pressures and abundance of oxygen atoms.

## Author Contributions

Author contributions: Y.L., Q.Z., S.N.W. and G.K. performed and analyzed calculations, Y.L., S.N.W. and A.R.O. wrote the paper, X.D. provided technical assistance with calculations.

## Figures and Tables

**Table 1:** Crystal structures of Al_4_O_7_ at 400 GPa and AlO_2_ at 500 GPa

Al_4_O_7_: Space group *C*2. Lattice parameters a = 4.598 Å, b = 9.670 Å, c = 5.094 Å, β = 153.50°				
	Wyckoff symbol	x	y	z
**Al1**	**4c**	0.2700	0.0001	0.5216
**Al2**	**2a**	0	0.2580	0
**Al3**	**2a**	0.5000	0.2859	0
**O1**	**2a**	0	0.4399	0
**O2**	**4c**	0.2624	0.1299	0.9110
**O3**	**4c**	0.2534	0.3140	0.5046
**O4**	**2b**	0.5000	0.1324	0.5000
**O5**	**2a**	0.5000	0.4568	0
AlO_2_: Space group *P*2_1_/*c*. a = 4.664 Å, b = 2.304 Å, c = 4.726 Å, β = 90.75°				
**Al**	**2a**	0.2217	0.2750	0.6315
**O1**	**2a**	0.1339	0.7582	0.8768
**O2**	**2a**	0.5016	0.1831	0.3849
